# Recent Advances in the Nutritional Screening, Assessment, and Treatment of Japanese Patients on Hemodialysis

**DOI:** 10.3390/jcm12062113

**Published:** 2023-03-08

**Authors:** Junko Ishida, Akihiko Kato

**Affiliations:** 1 Department of Food and Nutritional Environment, College of Human Life and Environment, Kinjo Gakuin University, Nagoya 463-8521, Japan; 2 Blood Purification Unit, Hamamatsu University Hospital, Hamamatsu 431-3192, Japan

**Keywords:** protein-energy wasting (PEW), nutritional screening, nutritional assessment, hemodialysis

## Abstract

Patients on hemodialysis (HD) have a higher rate of protein-energy wasting (PEW) due to lower dietary intake of energy and protein (particularly on dialysis days) and greater loss of many nutrients in the dialysate effluent than other patients. The most well-known method of nutritional screening is the subjective global assessment. Moreover, the Global Leadership Initiative on MalnutIrition has developed the first internationally standardized method for diagnosing malnutrition; however, its use in patients on HD has not been established. In contrast, the nutritional risk index for Japanese patients on HD has recently been developed as a screening tool for malnutrition in patients on HD, based on the modified PEW criteria. These tools are beneficial for screening nutritional disorders, enabling registered dietitians to assess patients’ dietary intake on dialysis and non-dialysis days and provide advice on dietary intake, especially immediately after dialysis cessation. Oral supplementation with enteral nutrients containing whey protein may also be administered when needed. In patients that experience adverse effects from oral supplementation, intradialytic parenteral nutrition (IDPN) should be combined with moderate dietary intake because IDPN alone cannot provide sufficient nutrition.

## 1. Introduction

Nutritional problems are prevalent in patients with chronic kidney disease (CKD). Malnutrition is closely associated with the risk of onset and progression of cardiovascular disease, sarcopenia, frailty, infection, and cognitive impairment. In addition, poor nutritional status is associated with poor healthy-longevity outcomes, such as the requirement of support and long-term care, institutionalization in nursing homes, and hospitalization [1]. Nutritional disorders in patients with kidney disease are termed as “protein-energy wasting” (PEW), a state of disordered catabolism resulting from metabolic and nutritional derangements. PEW is induced by both anorexia and decreased nutrient intake and various other factors such as uremic toxins, inflammation, oxidative stress, hyper-catabolism, metabolic acidosis, low testosterone levels, growth hormone resistance, insulin resistance, physical inactivity, loss of nutrients from urine and dialysate, and comorbidities [2,3]. Cachexia represents a very severe form of PEW that is often associated with profound physiological, metabolic, psychological, and immunological disorders [2].

Recently, the National Kidney Foundation and the Academy of Nutrition and Dietetics updated the National Kidney Foundation’s Kidney Disease Outcomes Quality Initiative (NKF-KDOQI) Clinical Practice Guidelines for Nutrition in Chronic Kidney Disease [4]. The guidelines suggest that patients with CKD stages 3–5D should be screened for nutrition status biannually. The guidelines also provide direction for when a registered dietitian should perform a detailed nutrition assessment. However, many of the statements in the guidelines were based on expert opinion from U.S. nephrologists. Furthermore, the cut-off values for nutritional parameters such as body mass index (BMI) and creatinine index in the guidelines cannot apply to Japanese patients on HD due to the smaller body size and muscle mass volume in this population. The timing of blood sampling before HD is also different between Japan (at 2-day intervals) and USA (mid-week), leading to inconsistencies in laboratory measures. Thus, an individualized and specialized approach will be needed to screen and assess the nutritional status of Japanese patients on HD.

The Dialysis Outcomes and Practice Patterns Study (DOPPS) reported that the prevalence of serum C-reactive protein (CRP) >10 mg/L was much lower in Japan (10%) than in seven European countries (30 to 44%) and Australia/New Zealand (36%), where 57% of CRP measurements were ≤1 mg/L in Japan [5]. In contrast, when the association of nutritional parameters was compared with mortality risk in 12 DOPPS-joining countries, the impact of malnutrition on total death was most potent in Japan [6]. It is therefore likely that the nutritional problem has more impact than microinflammation-induced catabolism in Japanese patients on HD upon improving their survival prognosis.

In this review, we mainly focus on inadequate nutritional intake in Japanese patients on HD. We first introduce the methods of nutritional screening and assessment for patients undergoing HD. Thereafter, we outline the relationship between malnutrition and sarcopenia and frailty and discuss the usefulness of the Global Leadership Initiative on Malnutrition (GLIM) criteria, which are proposed as the first international standard criteria for malnutrition diagnosis. Third, the daily micronutrient intake among patients on dialysis is often deficient. Particularly, magnesium (Mg) intake is independently associated with serum Mg level [7]. Deficiencies in the daily intake of zinc (Zn) is also related to worse nutritional and body composition parameters and higher mortality risk in patients on HD [8]. Thus, we review the effect of Mg and Zn deficiencies on the clinical outcomes of patients on HD [9,10]. In addition, we demonstrate the usefulness of oral nutritional support (ONS) and intradialytic parenteral nutrition (IDPN) as nutritional supplements for PEW in Japan.

## 2. Nutritional Assessment Methods for Dialysis Patients

The prevalence of PEW is high in patients on chronic HD and is closely associated with morbidity and mortality. The subjective global assessment (SGA) has been validated as an objective screening tool for nutritional risk in patients on chronic HD. Other screening tools include the malnutrition-inflammation score (MIS), geriatric nutritional risk index (GNRI), mini-nutritional assessment short form (MNA-SF), and malnutrition universal screening tool (MUST). Thus, we reviewed the usefulness of these screening tools for assessing nutritional risk in patients on HD.

### 2.1. SGA

The SGA is a nutritional assessment method that includes medical history and physical examination sections. The SGA is the most widely used method for assessing subjective nutritional status worldwide [11].

The medical history section comprises five questions on (1) weight loss (during the preceding 6 months or changes over the past 2 weeks), (2) dietary intake (compared to normal conditions), (3) gastrointestinal symptoms (over the last 2 weeks), (4) functional capacity or energy level, and (5) metabolic demands (relationship between disease and nutritional requirements). The physical examination section includes five questions on (1) loss of subcutaneous fat, (2) muscle wasting, (3) edema formation at the ankle or (4) sacrum, and (5) ascites. These criteria are subjectively evaluated as either normal (0), mild (1+), moderate (2+), or severe (3+). Based on medical history and physical examination findings, clinicians rank SGA severity into three categories: (A) well nourished, (B) moderate or suspected malnutrition, and (C) severe malnutrition [11].

In the United States, the 7-point SGA scale is used to assess the nutritional status of patients with CKD. This scale comprises six questions on weight change, dietary intake, gastrointestinal symptoms, functional capacity, disease status/comorbidities as related to nutritional needs, and physical examination (used to evaluate weight change such as loss of subcutaneous fat, muscle wasting, and edema related to undernutrition). Answers are rated from 1 to 7 points. A rating of 6 or 7 indicates very mild risk to no risk (well-nourished status), while 3 to 5 indicates mild to moderate risk, and 1 or 2 in most categories indicates significant physical signs of malnutrition.

Nutritional assessment using the 7-point SGA scale in elderly patients with advanced CKD (mean body mass index (BMI): 28.4 kg/m^2^) showed that 28% of the patients had moderate nutritional risk (SGA rating, 3 to 5) [12]. A study of 1601 Dutch patients on HD (mean age: 59 years) showed that 23% of the patients had moderate nutritional risk (SGA rating 4 to 5), and 5% had severe nutritional disorders (SGA rating, 1 to 3). Nutritional risk was also present in 55% of patients with BMI < 22 kg/m^2^, 40% of patients with BMI 22–25 kg/m^2^ (normal body weight), and 25% of patients with BMI > 30 kg/m^2^ (obesity). Lower BMI was associated with more frequent complications. In addition, the 7-point SGA was useful for predicting the 7-year mortality risk [13].

Therefore, the nutritional guidelines of the 2020 NKF-KDOQI recommend the use of the 7-point SGA for assessing the nutritional status of patients on dialysis (Level 1, Recommendation B) [4].

### 2.2. MIS

The MIS was initially developed by Kalantar-Zadeh et al. to assess nutritional deficits in patients on HD [8]. It comprises 10 items and a combination of the 7-point SGA with dialysis vintage, BMI, and laboratory parameters (serum albumin level and total iron-binding capacity) [14]. Each item is rated from 0 to 3 points, with a total score of 30 points, with higher scores indicating worse nutritional status. The KDOQI guidelines suggest MIS use as an assessment tool (Level 2, Recommendation C) [4]. A higher MIS score over time is associated with reduction in dietary intake, body fat percentage, upper-arm muscle circumference, and a high risk of hospitalization and mortality [14]. It has also been reported that the survival prognosis is poor when the MIS score is ≥7 [15]. Moreover, a global review demonstrated that 28% to 54% of patients on dialysis had nutritional risk when assessed using either the SGA or MIS [16].

### 2.3. GNRI

The GNRI was initially designed to predict the risk of malnutrition-related complications and mortality in elderly, hospitalized French persons [17]. The GNRI is a simple and objective tool for assessing nutritional status based on only actual and ideal body weight (BW) and serum albumin levels. Ideal BW can be calculated using the Lorentz formula, which considers a patient’s body height and sex. When body height measurement is difficult to perform, it is estimated using a formula based on knee height. When the actual BW exceeds the ideal BW, we set the actual/ideal BW ratio to 1 (Table 1) [18]. In Japan, BW corresponding to a BMI of 22 kg/m^2^ is often used as an ideal BW, and in patients on HD, dry weight is used as the actual BW [18].

The GNRI is frequently used for assessing nutritional conditions owing to its simplicity. However, this tool would likely reflect medium- to long-term nutritional status rather than short-term nutritional status because (1) the biological half-life of serum albumin is approximately 3 weeks, (2) albumin is mainly stored extravascularly, and (3) the variability of serum albumin level is less than its metabolic turnover rate. In addition, although the cut-off GNRI value for survival prognosis have been reported as 89.3 to 96.0 in patients on HD and 94.55 to 96.4 in patients on peritoneal dialysis (PD), there is no standard cut-off value for survival prognosis [19].

### 2.4. MNA-SF

The MNA-SF is a screening method for assessing the nutritional status of elderly individuals. The MNA-SF assesses six items to rate the nutritional state (0 to 14 points): (1) reduced dietary intake over the past 3 months (0 to 2 points), (2) weight loss over the past 3 months (0 to 3 points), (3) ambulation (0 to 2 points), (4) mental stress or acute illness over the past 3 months (0 to 2 points), (5) depression or dementia (0 to 2 points), and (6) BMI or calf circumference (0 to 3 points). A score of 7 or less indicates malnourishment, a score of 8–11 indicates the risk of malnutrition, and a score of 12–14 indicates a normal nutritional status [20].

When the MNA-SF was applied for patients on HD, 30.1% of the patients were classified as well nourished, 59.3% as being at risk of malnutrition, and 10.6% as malnourished [21]. In addition, there was a 2.50-fold higher risk of all-cause mortality in patients at risk of malnutrition and a 3.89-fold higher risk in malnourished patients compared with those with a normal nutritional status [21].

### 2.5. MUST

MUST is a nutritional screening method developed by the British Association for Parenteral and Enteral Nutrition for home-care patients. In MUST, assessment of nutritional risk is based on the total scores for BMI, weight loss, acute illness, and undernutrition (Table 2) [22]. In patients on HD, MUST can effectively screen for the presence of PEW, with a sensitivity of 100% and a specificity of 98% [23].

### 2.6. New Indicators for Muscle Wasting

Myostatin is a myokine predominantly expressed in skeletal muscle, and it regulates muscle growth negatively. Myostatin is overexpressed in uremic sarcopenia [24]. Recently, blood myostatin was reported to be negatively related to muscle mass and muscle strength, as assessed by handgrip strength, in patients on peritoneal dialysis [25] and in those on HD [26]. However, this association is not a universal finding. This may be due to the influence of various factors such as age, gender, inflammatory state, physical activity, and the different assay techniques for myostatin measurement [27].

Brain-derived neurotrophic factor (BDNF) is another myokine produced by immune cells and skeletal muscle. BDNF is involved in the regulation of synaptic function and in the maintenance of the neuromuscular system as well as in muscle development and metabolism [28]. Deus et al. [29] showed that 6 months of resistance training just before HD sessions improved handgrip strength in line with increased BDNF. Serum BDNF levels were also positively associated with handgrip strength, role-emotional, and emotional well-being scales and negatively associated with the Beck depression inventory score [29]. Miyazaki et al. [30] evaluated the relationship between BDNF and sarcopenia and frailty in regular patients on HD. Plasma BDNF levels were significantly lower in patients with severe sarcopenia and was correlated with muscle strength and physical performance, such as in the 6 m walk test, short physical performance battery, and the 5-time chair stand test. BDNF was also positively correlated with body weight. Similarly, in recipients of kidney transplantation, serum BDNF levels were significantly higher, but serum myostatin levels were significantly lower in the group with low skeletal muscle mass index (SMI), as measured with dual-energy X-ray absorptiometry (DXA), compared with the normal group [31].

Creatinine kinetic modelling is also proposed as an indirect indicator of muscle mass volume in patients on HD. The creatinine generation rate (CGR) can be calculated by measuring the pre- and post-dialysis creatinine concentrations. Because age and gender are independent determinants of CGR, CGR is usually adjusted for age and gender using a previously reported equation (i.e., %CGR) [32]. A recent study demonstrated that the cut-off value of %CGR for detecting low muscle mass volume (SMI less than 7.0 kg/m^2^ in men and less than 5.7 kg/m^2^ in women) was 109.83, with a sensitivity of 68% and a specificity of 88% in Japanese HD patients [33]. The ratio of serum creatinine to cystatin C is also demonstrated to be useful in predicting skeletal muscle mass and strength in patients with non-dialysis CKD [34].

## 3. Diagnostic Criteria for PEW

### 3.1. International Diagnostic Criteria

The International Society of Renal Nutrition and Metabolism (ISRNM) Expert Committee reported diagnostic criteria for PEW in 2008 (Table 3) [2]. The criteria comprised four categories. PEW is diagnosed when at least three out of the four categories (and at least one test in each of the selected category) are satisfied.

**Figure 1:**
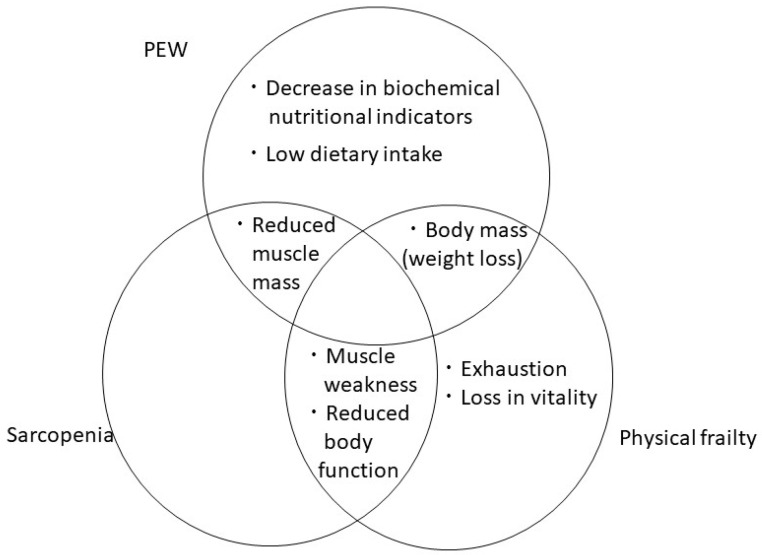
Association between protein-energy wasting (PEW) and sarcopenia and frailty.

PEW is caused by inadequate intake of nutrients and a hypercatabolic state in which skeletal muscle, visceral protein, and stored body fat are exhausted through various stimuli. The stimuli include inflammation, oxidative stress, accumulation of uremic toxins, insulin resistance, metabolic acidosis, and nutrient loss from effluent dialysate (Figure 1). In the category of low body mass, the BMI cut-off value was set at <23 kg/m^2^ [2]. However, since 74.1% of Japanese patients on HD have a BMI of <24 kg/m^2^, this cut-off is inappropriate for Japanese patients [35]. Therefore, the KDOQI guidelines state that diagnosing PEW using BMI is inappropriate unless the BMI is <18 kg/m^2^ [4].

### 3.2. Diagnostic Criteria for Japanese Patients on HD

The scientific committee of the Japanese Society for Dialysis Therapy (JSDT) developed a novel nutritional risk index, the Nutritional Risk Index for Japanese Hemodialysis (NRI-JH), after modifying the original PEW criteria (Table 4) [36]. The NRI-JH classifies patients on HD into three risk groups according to their total score, ranging from 0 to 13. The adjusted hazard ratio for 1-year survival was 1.96 (95% confidence interval (CI): 1.77–2.16) in the medium-risk group (score 8 to 10) and 3.91 (95% CI: 3.57–4.29) in the high-risk group (score 11 to 13), with the low-risk group (score 0 to 7) serving as a reference. The NRI-JH can stratify mortality risk in elderly patients on HD [36]. It is also useful for predicting long-term mortality [37].

## 4. Association of Malnutrition with Sarcopenia and Frailty

Malnutrition and sarcopenia/frailty are bidirectionally associated, but few studies have assessed the relationship between sarcopenia/frailty and nutritional status in the dialysis population.

### 4.1. SGA

Complications of nutritional risk by the 7-point SGA (≤5 points) in patients on HD were found in 66.7% of patients with sarcopenia, 65.7% of patients with pre-sarcopenia, and 51.2% of patients without sarcopenia [38]. There was a 2.99-fold higher risk of all-cause mortality when the patients had complications of malnutrition and sarcopenia, indicating that sarcopenia and malnutrition may additively worsen survival prognosis [38].

### 4.2. MIS

The MIS was inversely correlated with muscle power as assessed by handgrip strength in patients on HD (mean age: 58.3 years). The MIS was significantly associated with the risk of low handgrip strength (below the cut-off value for sarcopenia; odds ratio (OR) 1.202; 95% CI 1.073–1.347; *p* < 0.01) and with mortality (OR 1.322; 95% CI 1.192–1.467; *p* < 0.01), indicating that a worse nutritional status increases the risk of sarcopenia and mortality [39]. Among patients on PD, there was a significantly higher MIS in those with physical frailty than in those without (7.13 ± 3.22 vs. 5.12 ± 2.30, *p* < 0.01). In addition, patients with physical frailty and depressive symptoms had worse MIS scores (9.48 ± 3.97) [40].

### 4.3. GNRI

In Japanese patients on HD, the GNRI cut-off value for mortality (=91.5) [18] was related to the risk of a handgrip strength below the cut-off value associated with sarcopenia (based on the criteria of the Asian Working Group for Sarcopenia (AWGS) 2019 (male < 28 kg, female < 18 kg)) [41]. However, the sensitivity of the cut-off value for GNRI indicating possible sarcopenia by handgrip strength was 46%, and the specificity was 61% [41]. In addition, there was no difference in GNRI according to frailty status in Japanese patients on HD [42]. Therefore, further studies are needed to clarify the cut-off value of GNRI for the early detection of sarcopenia and frailty.

### 4.4. MNA-SF

Among malnourished patients on HD (MNA-SF score: 0 to 7), 43.5% of the patients were frail, and 34.8% were pre-frail. In addition, risk of malnutrition (MNA-SF score: 8 to 11) was complicated by frailty and pre-frailty in 30.1% and 50.0% of the patients, respectively. In contrast, frailty was found in only 12.4% of the patients with a normal nutritional status (MNA-SF score: 12 to 14) [40]. Patients on HD with MNA-SF ≤ 11 also had a 7.1-fold higher risk of frailty than those with good nourishment [42].

### 4.5. PEW

Muscle mass loss is included in the diagnostic criteria for PEW and sarcopenia. Body weight loss is also a common criterion for PEW and physical frailty (Figure 1). Therefore, PEW is expected to be closely related to sarcopenia and frailty.

### 4.6. GLIM Criteria

**Figure 2:**
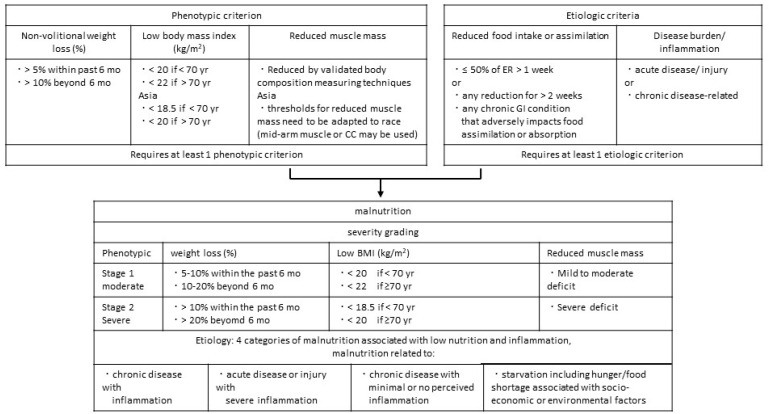
Malnutrition diagnosis using Global Leadership Initiative on Malnutrition (GLIM) standards. Abbreviations: CC, calf circumference; ER, emergency requirements; GI, gastrointestinal; mo, month; yr, year. Modified from [43].

The GLIM criteria are the first internationally standardized diagnostic criteria for malnutrition in adults. They are applied using a two-step method involving risk screening and diagnostic assessment. First, nutritional risk screening is performed using validated screening tools (MNA-SF, SGA, MUST, GNRI, etc.). If the patient is considered at risk of malnutrition, the presence and severity of malnutrition are evaluated based on two categories: “phenotypic” and “etiologic” [43]. Body weight loss, reduced BMI, and reduced muscle mass are categorized as phenotypic criteria, whereas reduced food intake/assimilation and disease burden/inflammation are classified as etiologic criteria. For the diagnosis of malnutrition, the GLIM recommends the use of a combination of at least one phenotypic criterion and one etiologic criterion (Figure 2). Severity grading is determined using these three phenotypic items. If at least one of the criteria was classified as moderate or severe, we diagnosed moderate or severe malnutrition.

The criteria for loss of muscle mass were not described in the GLIM categories. However, in elderly Japanese inpatients, mild to moderate muscle mass loss corresponds to a maximal calf circumference of ≤30 cm in men and ≤29 cm in women, and severe loss corresponds to ≤28 cm in men and ≤26 cm in women [44]. Therefore, muscle mass loss can be substituted by calf circumference measurement.

Presently, the usefulness of the GLIM criteria for diagnosing malnutrition in patients on dialysis is unclear. A recent study demonstrated that the sensitivity of the GLIM criteria in detecting malnutrition diagnosed by a well-established method (either 7-point SGA or MIS) was low (61 to 72%), indicating that the GLIM score did not perform better than the 7-point SGA and MIS. In addition, its predictive ability for survival prognosis was inferior to that of the 7-point SGA and MIS [45]. In contrast, a Korean study showed that malnutrition diagnosed using the GLIM criteria was significantly associated with the risk of all-cause mortality and hospitalization due to infection in patients on chronic HD [46].

## 5. Anorexia and Nutritional Deficiencies in Dialysis Patients

### 5.1. Anorexia

Approximately 40% of patients on HD were aware of loss of appetite when asked about their appetite in the past 4 weeks [47]. In particular, appetite tended to decrease during lunch and dinner on dialysis days. The suggested causes of anorexia are (1) suppression of appetite signaling to the hypothalamus by increased inflammatory cytokines; (2) increased tryptophan transport across the blood–brain barrier due to decreased blood levels of branched-chain amino acids, which enhance serotonin synthesis in the brain and suppress appetite; (3) decreased ghrelin production from gastric endocrine cells with appetite-promoting effects; and (4) dysgeusia due to poor oral environment or zinc deficiency.

### 5.2. Insufficient Nutritional Intake

Achieving adequate dietary energy and protein intake remains a challenge for patients on HD. A recent review including eight studies with more than 100 patients on HD reported that dietary energy inadequacy (<35 kcal/kg BW/day) was found in 52 to 92% of the patients, whereas dietary protein inadequacy (<0.8 g/kg BW/day) was found in 32.3 to 81% [48].

### 5.3. Nutrient Loss during Dialysis

Approximately 6–12 g of amino acids and 7–8 g of protein are lost during each dialysis session [48]. In addition, water-soluble vitamins and trace elements such as zinc and carnitine are removed by diffusion and filtration. Furthermore, online hemodiafiltration removes more water-soluble vitamins and larger protein molecules than conventional HD [49].

### 5.4. Zn Deficiency

Because Zn is a trace element that is essential for maintaining the structure and functional expression of many proteins including enzymes, various symptoms occur in its deficiency. Serum Zn levels decrease with the progression of CKD stage, and approximately 70% of patients on HD and 60% of patients on PD have Zn deficiency (blood Zn < 60 μg/dL) [50].

Zn deficiency induces inflammation in muscle cells and increases oxidative stress by decreasing the activity of Zn-dependent antioxidant enzymes. In addition, Zn deficiency may contribute to the onset and progression of sarcopenia by decreasing the synthesis and increasing the degradation of muscle proteins, destabilizing neuromuscular transmitter sites and impairing neurotransmitter release. Zn deficiency also damages taste bud cells, leading to taste abnormalities.

In patients with CKD, there is a negative correlation between serum Zn concentration and salt taste-perception threshold; thus, the amount of daily salt intake is higher in Zn-deficient patients [51]. In addition, patients on HD with inadequate Zn intake (men < 10, women < 8 mg/day) had a 4.1-fold higher risk of all-cause mortality than those with adequate Zn intake [52].

**Figure 3:**
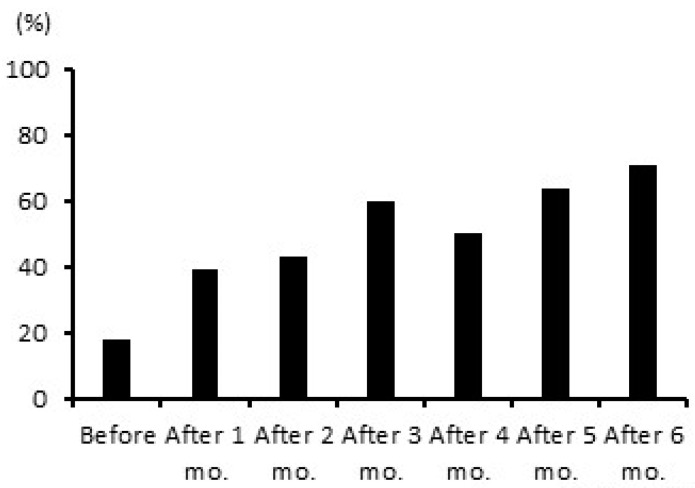
Taste thresholds during 6 months of zinc replacement therapy. Subjects: Twenty-eight patients on hemodialysis with serum zinc levels < 80 μg/dL (male: 19, female: 9). Methods: Nobelzin^®^ tablets (zinc acetate dihydrate) were orally administered to maintain serum zinc levels within 70–120 μg/dL, and salty taste thresholds were measured with the “SALSAVE^®^ impregnated paper test”. Those who could taste salt in the salt-impregnated filter paper with the lowest salt concentration were considered to have normal taste, the percentage of whom is shown.

In the general population, the relationship between blood Zn levels and dietary Zn intake was weak [53,54], although it is known that an association exists between zinc deficiency and reduced taste thresholds [55]. Similarly, there was no relationship between serum Zn and dietary Zn intake in patients on HD [56]. However, oral Zn supplementation is useful for mitigating taste disorders in patients on HD [56,57]. For example, a dose of Zn acetate (50 mg/day) for 6 months improved taste sensations such as salty, sweet, and bitter along with an increase in serum Zn concentration from 75 ± 8 to 97 ± 10 μg/dL [58]. We also preliminary observed improvement in salty taste thresholds in 28 patients on HD following oral Zn administration for 6 months (Figure 3, unpublished data). However, long-term Zn acetate administration should be avoided because Zn can directly prevent copper absorption from the intestinal tract, which may cause leukopenia and pancytopenia due to severe copper deficiency [59].

### 5.5. Mg Deficiency

Mg is the second most abundant intracellular cation. It performs various functions such as membrane stabilization, nerve conduction, ion transport, and intracellular energy metabolism and is involved in all reactions that require adenosine triphosphate (ATP). Mg is abundant in seaweed, seafood, sesame seeds, and nuts and is absorbed through the small intestine. Hypomagnesemia (<1.8 mg/dL) is one of the most common electrolyte disorders. The prevalence of hypomagnesemia did not decline even in CKD stages G4 and G5, where the prevalence rate was approximately 15% [60]. Since potassium-rich foods are rich in Mg, dietary restriction of potassium may lead to a lower intake of Mg. Moreover, hypomagnesemia may be induced by proton pump inhibitors, which are known to inhibit the passive/active transport of Mg from the small intestine [61].

Mg inhibits the formation of calcium protein particles (CPP) associated with the development of vascular calcification [62]. Among patients on HD, there is a J-curve relationship between hyperphosphatemia and mortality in patients with serum Mg concentration < 2.7 mg/dL, while the risk disappeared in patients with serum Mg > 3.1 mg/dL, implying that Mg may protective against hyperphosphatemia [62].

In patients on HD, Mg can be supplied by increasing the Mg concentration in the dialysate or administering oral Mg preparations. When the Mg concentration of the dialysate was increased from 1 to 2 mEq/L, secondary CPP formed after a significantly long period (after 28 days), suggesting that Mg supplementation may slow vascular calcification [63]. A meta-analysis of eight studies demonstrated that oral Mg supplementation also decreased serum parathyroid hormone levels and the intima-media thickness of the common carotid artery [64].

### 5.6. Oral Dysfunction

Oral function declines with age. Oral dysfunction is closely related to poor food intake, which can easily lead to dysphagia and malnutrition. Oral problems also affect the development of sarcopenia and frailty [65,66,67].

In Japan, a large-scale cohort study on the oral condition of elderly individuals was conducted in 2012, with a follow-up study lasting up to 4 years. The study reported that decline in ≥3 of six oral indicators (number of remaining teeth, chewing ability, tongue movement, gliding tongue, increase in food not chewed, and munching) was associated with a higher risk of physical frailty, sarcopenia, admission to nursing care, and death. Therefore, the appearance of such trivial deterioration in oral function is called “oral frailty” [68]. In 2018, “oral hypofunction” was added to Japan’s healthcare fee list. Oral cavity dysfunction is evaluated by seven examination items: poor oral hygiene, oral dryness, reduced occlusal force, decreased tongue-lip motor function, decreased tongue pressure, decreased masticatory function, and deterioration of swallowing function (Table 5). Thus, oral hypofunction is defined as a state where three or more of these diagnostic criteria are met [69]. The Japanese Society of Gerodontology Academic Committee classified the process from health to oral dysfunction into four stages: the 1st stage of population approach, the 2nd stage of oral frailty, the 3rd stage of oral hypofunction, and the 4th stage of oral dysfunction. In addition, “oral frailty” is widely used in public campaigns to raise awareness regarding oral function.

Although patients on dialysis experience oral diseases more frequently than healthy individuals, dental care is limited. We preliminarily assessed the presence of sarcopenia, based on the AWGS 2019 cut-off values and oral hypofunction, in 141 patients on HD. We found that there was no difference in oral hygiene and swallowing function between the sarcopenia and non-sarcopenia groups, while the other items were significantly lower in the sarcopenia group (Table 6, unpublished data). Since oral frailty is detected at the pre-frail stage, it is important to objectively evaluate the oral condition and preserve oral function to prevent the development of PEW, sarcopenia, and frailty.

## 6. Nutritional Supplementation for PEW

### 6.1. Meal Supply at Dialysis Facilities

As large amounts of protein and amino acids are lost during HD sessions, it is reasonable to encourage patients to eat meals during or just after the dialysis procedure. Comparative studies showed that dietary energy and protein intake were lower during the dialysis-on days than during the dialysis-off days, indicating that in-central patients on HD may skip three meals per week during dialysis treatment. Therefore, the supply of intradialytic meals may be a therapeutic opportunity to improve PEW and health-related quality of life, particularly in malnourished patients, unless there is a low risk of postprandial hypotension, digestive symptoms, or aspiration. Recently, it was reported that the intake of milk protein (40 g) 1 h after the start of dialysis compensates for amino acid loss from the dialysate effluent and maintains plasma amino acid concentrations until the end of the dialysis session [70].

Many dialysis facilities have stopped providing meals due to the ongoing coronavirus disease (COVID-19) pandemic in Japan. A retrospective study demonstrated that because of the discontinuation of meal provision for 10 months, dry weight gradually decreased from 53.6 to 52.6 kg, and GNRI decreased from 91.5 to 89.5 in elderly patients on HD [71].

### 6.2. Home Nutrition Care for Patients on Dialysis

In patients on dialysis, weighted mean adherence rates of the recommended energy, protein, and fat intakes were 23.1%, 45.5%, and 41.4%, respectively [72]. Below are some tips for increasing energy and protein intake at home:

1. Increase energy intake: Fat contains high energy (9 kcal/g); thus, dietary fat intake is useful for increasing energy levels. Dietary fat is present in oils, fatty meats, dairy products, nuts, etc., which are present in beef or pork ribs, chicken thighs with skin, bacon, fried tofu, and cream cheese eaten with bread. In particular, medium-chain triglycerides (MCTs) are useful as energy sources because they do not form micelles, enter the general circulation system rapidly through the portal vein, and are transported to the liver for β-oxidization. Octane, a medium-chain fatty acid, activates ghrelin, which has appetite-promoting effects. In a study on healthy subjects, an intake of 45 mL/day of MCT oil increased the blood levels of active ghrelin by approximately two-fold [73]. Since MCT oil is tasteless and odorless, it can be easily added to main foods and side dishes;
2. Increase in protein intake: To increase protein intake, it is crucial to eat well on dialysis day. Eating a meal at the time of dialysis visit improves nutritional status and quality of life and reduces inflammatory reactions and mortality [74]. Although proteins are abundant in meat, fish, eggs, beans, and dairy products, it is important to consume a well-balanced diet rather than one rich in only one food group. In elderly Japanese patients on HD (70 years or older), amino acid supplementation may also be useful because oral administration of 12 g/day of amino acid preparations for 6 months improves appetite and increases protein intake and body weight [75].

### 6.3. Oral Nutritional Supplement: ONS

When dietary counseling is insufficient to achieve the planned nutritional requirements, ONS is recommended as the first step of nutritional support for patients on HD. ONS can add up to 10 kcal/kg and 0.3–0.4 g of protein/kg daily to spontaneous intake, helping the achievement of nutritional targets [76]. Intradialytic intake of protein-rich food or oral supplements appears to be effective in mitigating the catabolism associated with hemodialysis procedures and increasing the total protein intake.

ONS should be initiated with a daily protein intake of >1.2 g/kg/day [76]. The goals of ONS are achieving (1) serum albumin > 4.0 g/dL, (2) serum transthyretin (prealbumin) > 30 mg/dL, and (3) energy intake > 30–35 kcal/kg/day [76]. The K/DOQI guidelines [4] recommend that ONS be continued for at least 3 months. A meta-analysis of previous studies found that ONS increased serum albumin by an average of 0.22 g/dL in patients on HD [77].

Whey protein has several advantages such as fast absorption speed, high body retention rate, and a 26% content of branched-chain amino acids (14% leucine). In patients on PD, daily intake of whey protein powder (27.4 g, 116 kcal) ensured target protein intake and improved body indices such as body weight and body composition [78]. In patients on HD, an oral intake of 15 g of whey protein (plus 6000 IU of vitamin E) weekly for 8 weeks improved SGA and MIS [79]. Thus, enteral nutrients containing whey proteins are useful nutritional supplements.

A randomized clinical trial (RCT) was conducted in patients on HD, without diabetes, and with energy intakes of <30 kcal/kg/day. The subjects were divided into the two groups: one group ingested fat mainly from ONS (300 kcal, 97% energy from fats) added once daily after a meal, and the other group consumed only the routine diet [80]. After 12 weeks, although BMI, serum albumin, and body fat ratio increased slightly with the addition of ONS, the bioelectrical impedance analysis-derived phase angle did not differ between the two groups, indicating that energy supplementation with lipids is nutritionally inadequate.

### 6.4. IDPN

If nutritional requirements cannot be met with meals or ONS, IDPN should be considered [81]. IDPN is usually administered for 4 h throughout the dialysis session thrice a week. However, IDPN during dialysis three times a week cannot provide sufficient nutritional requirements; therefore, patients with IDPN should receive at least energy ≥ 20 kcal/kg/day and protein ≥ 0.8 to 0.9 g/kg/day from meals. With the revision of the package insert in June 2020, patients on dialysis in Japan can also use amino acid infusion preparations for general use and liver failure as well as general-use kit infusion products containing amino acids and glucose [81]. If oral intake combined with IDPN does not provide the required nutrients, total parenteral nutrition (TPN) should be considered.

Because of the risk of hyperglycemia, IDPN begins with a low concentration of glucose and then changes to a higher concentration after confirming that blood glucose levels do not increase. According to the guidelines of the European Society for Clinical Nutrition and Metabolism (ESPEN), the first week should be started at no more than 8 mL/kg/IDPN (500 mL at 60 kg) and up to 16 mL/kg/IDPN at the maximum, and then, the dosage per dialysis should not exceed 1000 mL [82]. In a recent RCT on patients with HD, IDPN was administered to a group of patients who had adequate dietary intake (energy ≥ 20 kcal/kg/day, protein ≥ 0.8 g/kg/day) but were unable to continue ONS due to digestive symptoms [83], while the control group only received weekly nutritional counseling. At three months, serum albumin increased from 3.6 to 3.8 g/dL, and energy intake and body weight increased in the IDPN group (total energy: 1100 kcal, water: 986 mL, continuous administration over 4 h on dialysis) compared to the control group. Therefore, IDPN may be useful in patients who cannot continue ONS because of their digestive symptoms.

### 6.5. New Medical Treatments

Ghrelin is an endogenous hormone that decreases acute and chronic inflammation, enhances the immune system, stimulates appetite, and causes physiologic pulsatile release of GH. Low ghrelin values in HD patients with PEW are linked to a markedly increased mortality risk, especially due to cardiovascular causes [84]. Because these wasted patients are so anorectic, ghrelin therapies may be useful in the treatment of PEW. A randomized crossover double-blind study [85] demonstrated that treatment with an oral ghrelin agonist, MK-0677, for 30 days provided a positive effect on IGF-1 in patients in HD. Anamorelin hydrochloride, a ghrelin receptor agonist, was firstly approved for gastric, pancreatic, and colorectal cancer patients with cachexia in Japan. Thus, this oral ghrelin agonist is expected to bring new advancements into the field of clinical nutrition as an effective therapeutic drug for cachexia.

Chronic *Helicobacter pylori* (*H. pylori*) infection in the gastric mucosa is associated with abnormal ghrelin levels. Therefore, eradication of *H. pylori* by proton-pump inhibitor and antibiotics may be useful to mitigate the progress of gastric atrophy and prevent a decline of plasma ghrelin and subsequent PEW [86].

Myostatin is an important therapeutic target for treating CKD-related sarcopenia. There are two strategies to inhibit myostatin pathways: one is a blockade by direct binding to myostatin itself, and the other is inhibition of the myostatin–ActRIIB complex. However, clinical studies in sarcopenic patients demonstrated that the anabolic intervention is likely better if a block of ActRII receptors is used. It is also becoming clear that myostatin-targeted therapies should not be seen as a substitute for physical activity and nutritional supplementation [87].

Insulin has a critical role in both glucose metabolism and in the maintenance of skeletal muscle mass. Activation of dipeptidyl peptidase 4 is associated with impairment of insulin signaling in skeletal muscle, presumably leading to loss of muscle function. Therefore, dipeptidyl peptidase 4 inhibitors (DPP4-I) are good candidates for sarcopenia treatment. In elderly patients with type 2 diabetes, the DPP4-I group showed greater muscle mass as well as better muscle strength and physical performance as compared with the sulfonylurea group [88]. A retrospective observational study also demonstrated that a DPP-4 inhibitor prevented the progressive loss of muscle mass, as evaluated by DXA, with ageing in elderly diabetic patients [89]. Sencan et al. [90] also found out that adding a DPP-4 inhibitor to the patients’ treatments could effectively and significantly result in a positive effect on muscle strength during a 6-month follow-up period.

DPP-4 inhibitors are available for HD patients. Although there was no report to test the efficacy of DPP4-I on sarcopenia in advanced CKD patients, this agent may be a candidate in the prevention of muscle mass loss in diabetic patients on HD.

## 7. Conclusions

In this paper, we show a novel nutritional screening and assessment tool for Japanese patients on HD. The NRI-JH, a composite score of BMI, serum creatinine, albumin, and total cholesterol, is useful for screening the nutritional risk of Japanese patients on HD. When the total NRI-JH score exceeds 8 of 13 points, it is important to apply the GLIM criteria to diagnose the presence of malnutrition. Zn deficiency is very common in patients on HD and is associated with a lower threshold of salty taste. In addition, Mg deficiency is related to the progression of vascular calcification. Thus, when a patient on HD is diagnosed with malnutrition, Zn and Mg deficiencies should be evaluated.

When poor dietary intake is likely to be associated with the development of PEW, the first step is to review the daily diet and, if necessary, start ONS with enteral nutritional supplements. Enteral nutrition, particularly including whey proteins, is beneficial. Although IDPN is useful for nutritional supplementation in patients on HD who can eat to some extent, it should always be used in combination with oral intake of food or ONS since IDPN alone cannot replace deficient nutrients.

## Figures and Tables

**Table 1:** Geriatric Nutritional Risk Index (GNRI).

GNRI formula	(14.89 × albumin (g/dL)) + (41.7 × (weight/ideal weight)) ideal weight is calculated from the Lorentz equations (WLo) or weight equivalent to BMI = 22 kg/m^2^
The Lorentz equations (WLo) formula	For men: ideal weight = H − 100 − ([H − 150]/4) For women: ideal weight = H − 100 − ([H − 150]/2.5)
If height cannot be obtained	For men: H (cm) = (2.02 × KH (cm)) − (0.04 × age (y)) + 64.19 For women: H (cm) = (1.83 × KH (cm)) − (0.24 × age (y)) + 84.88

Modified from [18]. Abbreviations: albumin, serum albumin; weight, actual body weight; ideal weight, ideal body weight; H, height; KH, knee height. NOTE: When the body weight exceeds the ideal body weight, the ideal weight rather than the actual weight is used to calculate the index. In the original reference, four grades of nutritional-related risk are defined: major risk (GNRI: <82), moderate risk (GNRI: 82 to <92), low risk (GNRI: 92 to ≤98), and no risk (GNRI: >98). For patients on hemodialysis: risk of nutrition (GNRI ≤ 91), without risk (GNRI > 91).

**Table 2:** Malnutrition Universal Screening Tool (MUST) diagnostic criteria.

Score	BMI (kg/m^2^)	Unplanned Weight Loss in the Past 3–6 Months (%)	Acute Disease Effect + No Nutritional Intake for > 5 Days
0	>20.0	<5	None
1	18.5–20.0	5–10	
2	<18.5	>10	There has been or is likely

Modified from [22]. Total score of 6 points: 0, low risk (routine clinical care); 1, medium risk (observe); ≥2, high risk (active intervention of dietitian or nutrition support team).

**Table 3:** Protein energy wasting (PEW) diagnostic criteria.

Category	Criteria
Serum chemistry	Serum albumin < 3.8 g per 100 mL (bromocresol green test) Serum prealbumin (transthyretin) < 30 mg per 100 mL (for patients on hemodialysis alone) Serum cholesterol < 100 mg per 100 mL
Body mass	BMI < 23 kg/m^2^ (Asians have low BMI) Unintentional weight loss: 5% over 3 months or 10% over 6 months Total body fat percentage < 10%
Muscle mass	Reduced muscle mass: 5% over 3 months or 10% over 6 months Reduced mid-arm muscle circumference area: reduction > 10% In relation to 50th percentile of reference population Creatinine appearance
Dietary intake	Unintentional low DPI: <0.8 g/kg/day for at least 2 months for patients on dialysis or <0.6 g/kg/day for patients with CKD stages 2–5 Unintentional low DEI: <25 kcal/kg/day for at least 2 months

Modified from [2]. Abbreviations: DPI, dietary protein intake; DEI, dietary energy intake. NOTE: At least three out of the four listed categories (and at least one test in each of the selected categories) must be satisfied for the diagnosis of kidney disease-related PEW. However, these diagnostic criteria have not been widely used because body composition and dietary intake need to be assessed over several months, and the validity of the cut-off values for Japanese patients with CKD is unknown.

**Table 4:** NRI-JH evaluation method.

Category	Criteria	Score
BMI	• ≥20 kg/m^2^	3
	• <20 kg/m^2^	0
Serum albumin (BCG)	• Age ≥ 65, <3.5 g/dL, age < 65, <3.7 g/dL	4
	• Age ≥ 65, ≥3.5 g/dL, age < 65, ≥3.7 g/dL	0
Serum creatinine	• Age ≥ 65, male < 9.7 mg/dL, female < 8.0 mg/dL	4
	• Age ≥ 65, male ≥ 9.7 mg/dL, female ≥ 8.0 mg/dL	0
	• Age < 65, male < 11.6 mg/dL, female < 9.7 mg/dL	4
	• Age < 65, male ≥ 11.6 mg/dL, female ≥ 9.7 mg/dL	0
Serum total cholesterol	• <130 mg/dL	1
	• ≥130 to <220 mg/dL	0
	• ≥220 mg/dL	2

Modified from [36]. Abbreviations: BCG, Bromocresol Green. NOTE: the sum of each point was calculated and divided into three risk groups: low-risk group (score, 0–7), medium-risk group (8–10), and high-risk group (11–13). BMI was calculated from weight measured after hemodialysis, and laboratory data were measured before hemodialysis.

**Table 5:** Measurements of clinical signs/symptoms of oral hypofunction.

Clinical Signs	Measurements
Poor oral hygiene	The total number of microorganisms (CFU/mL) is ≥106.5, or the revised tongue coating index is ≤50%
Oral dryness	The measured value obtained by a recommended moisture checker is less than 27.0
Reduced occlusal force	The occlusal force is less than 200 N, or the number of remaining teeth is <20
Decreased tongue-lip motor function	The number of any counts of /pa/, /ta/, or /ka/ produced per second is <6
Decreased tongue pressure	The maximum tongue pressure is less than 30 kPa
Decreased masticatory function	The glucose concentration obtained by chewing gelatin gummies is <100 mg/dL
Deterioration of swallowing function	The total score of the Eating Assessment Tool (EAT-10) is ≥3

Modified from [69].

**Table 6:** Association between sarcopenia and oral hypofunction.

Oral Hypofunction	Dysfunction-Criteria	Sarcopenia		*p*
		Sarcopenia Groups	Non-Sarcopenia Groups	
Poor oral hygiene *^1^ (%)	≥50	17 (0–89)	22 (0–67)	N.S.
Oral dryness *^2^	<27	25 (6–38)	26 (17–70)	<0.05
Reduced occlusal force (teeth number) *^3^	<20	16 (0–31)	24 (0–32)	<0.05
Decreased tongue-lip motor function *^4^ (times/sec)	Any of them <6	5 (2–7)	6 (1–8)	<0.05
Decreased tongue pressure (kPa)	<30	25 (7–48)	33 (15–55)	<0.05
Decreased masticatory function *^5^ (mg/dL)	<100	106 (22–266)	124 (25–340)	<0.05
Deterioration of swallowing function *^6^ (point)	≥3	0 (0–10)	0 (0–32)	N.S.

Subjects: A total of 141 patients on hemodialysis (male: 96, female: 45). Variables were expressed as the median (range). Data were analyzed by the Mann–Whitney U-test. Note: *^1^ The measured revised tongue coating index (TCI); *^2^ the measured value obtained by a recommended moisture checker; *^3^ number of remaining teeth; *^4^ the number of any counts of /pa/, /ta/, or /ka/; *^5^ the glucose concentration obtained by chewing gelatin gummies; *^6^ the total score of the Eating Assessment Tool (EAT 10) questionnaires.

## Data Availability

Not applicable.
